# The Medicinal Plant Pair *Bupleurum chinense*-*Scutellaria baicalensis* – Metabolomics and Metallomics Analysis in a Model for Alcoholic Liver Injury

**DOI:** 10.3389/fphar.2019.00254

**Published:** 2019-03-20

**Authors:** Zefang Dang, Qianhua Li, Shujun Sun, Yang Wang, Rui Lin, Yongyu Zhang, Jianye Dai, Ningning Zheng

**Affiliations:** ^1^School of Pharmacy, Shanghai University of Traditional Chinese Medicine, Shanghai, China; ^2^Institute for Interdisciplinary Medicine Sciences, Shanghai University of Traditional Chinese Medicine, Shanghai, China; ^3^Frontier Medical Service Brigade, Army Medical University, Hutubi, China; ^4^School of Traditional Dai-Thai Medicine, West Yunnan University of Applied Sciences, Jinghong, China; ^5^School of Pharmacy, Lanzhou University, Lanzhou, China

**Keywords:** metabolomics, metallomics, *Bupleurum chinense*-*Scutellaria baicalensis*, herbal pair, alcoholic liver injury

## Abstract

Traditional Chinese Medicine (TCM), a complex natural herbal medicine system, has increasingly attracted attention from all over the world. Most research has illustrated the mechanism of TCM based on the active components or single herbs. It was fruitful and effective but far from satisfactory as it failed to gain insights into the interactivity and combined effects of TCM. In this work, we used *Bupleurum chinense* (*B. chinense* DC, a species in the genus *Bupleurum*, family *Apiaceae*) and *Scutellaria baicalensis* (*S. baicalensis* Georgi, a species in the genus *Scutellaria*, family *Lamiaceae*), an herbal pair in TCM, to illustrate the combined effect. We compared the diverse effects between the *B. chinense-S. baicalensis* herbal pair and its compositions in an animal model of Alcoholic Liver Injury to highlight the advantages of the formula. Biochemical and histological indicators revealed that the effect of *B. chinense-S. baicalensis* was better than its individual parts. Furthermore, metabolite profiling of the serum, liver tissue, and feces were conducted to reveal that the herbal pair largely presented its effects through enhanced tissue penetration to maintain liver-located intervention with less global and symbiotic disturbance. Furthermore, we analyzed the distribution of the metal elements in extracts of the serum and liver tissue and found that the herbal pair significantly regulated the distribution of endogenous selenium in liver tissue. As selenium plays an important role in the anti-oxidative and hepatoprotective effects, it may be the reason for combined effects in BS formula. This research could open new perspectives for exploring the material basis of combined effects in natural herbal medicine.

## Introduction

Traditional Chinese Medicine, a complex medical system with a long history of usage, has increasingly been examined. Intervention in the human body by compound herbal formulas is one of the major characters of TCM. So far, many endeavors have been made to unveil the mechanisms of TCM by way of the separation strategy, which is to study the pharmacodynamic material basis by tapering the entire formula to single herbs, even to single active components. However, study of the complexity of TCM requires a different strategy as the separation strategy may lose sight of the interactivity and combined effects (Zheng et al., 2013). In fact, combination therapies have been increasingly utilized during the past few years (Yohannes et al., 2010). A multi-drug strategy usually yields more therapeutic efficacy with less adverse effects, which may lead to a triumph for modern medicine and provide fertile ground for modern drug development (Lee et al., 2011; Wang et al., 2013). In particular, the herbal pair, the minor formula composed of two herbs with distinct properties, is used by clinical TCM practitioners to increase the therapeutic effect or reduce toxic effects (Li et al., 2010). To investigate the integration mechanism of the formula, technologies with holistic concepts are needed (Cappuccio et al., 2015). Recently, systems biology combined with physiology, biochemistry, molecular biology and tongue image digitization were employed to evaluate the treatment effect and explore the mechanisms of the TCM formula (Wu et al., 2010; Dai et al., 2013a; Sun et al., 2013). The focus has been on the constituent changes and chemical transformations to explain the potential combined effect of the TCM formula (Li et al., 2010; Ye et al., 2011; Cao et al., 2014; Zhang Y. et al., 2016). Furthermore, metal or semi-metal elements have attracted our attention for the numerous physiological and biochemical functions, thus helping researchers to better illustrate the complex pharmacodynamic mechanism of TCM (Dong et al., 2006; Yan et al., 2015; Deng et al., 2018).

*Bupleurum chinense-Scutellaria baicalensis* (BS), the simultaneous usage of *B. chinense* and *S. baicalensis*, was originally recorded in the ancient Chinese medical book Shanghan Lun (Treatise on Cold Damage Diseases). It is one of the most common herbal pairs used in clinical trials to treat hepatic disease (Lin et al., 2011; Wang et al., 2014). It is considered as being able to achieve better effects with less toxicity, yet the in-depth mechanism is unclear. Physiological and biochemistry studies were conducted to highlight the advantages of the formula in an ALI animal model. Then, systematic metabolomics combined with metallomics were further employed to illustrate the precise regulation of disease-intervened metabolites and trace element distribution in liver tissue, which may explain the combined effects present in the BS formula. Our research not only presented the systematic comparison of the pharmacodynamic effects between herbal pairs and its compositions, but also provided new perspective for exploring the material basis of the formula’s combined effect.

## Materials and Methods

### Materials and Reagents

Analytical grade methoxyamine hydrochloride, methanol, ethanol, chloroform, and pyridine were obtained from China National Pharmaceutical Group Corporation in Shanghai of China. *N*,O-Bis(trimethylsilyl)trifluoroacetamide (BSTFA:TMCS, 99 : 1), heptadecanoic acid and urease were purchased from Sigma-Aldrich. Liquor (56 %, v/v, 00215) was obtained from Beijing Red Star, China. Double-distilled water was produced by a Milli-Q Ultra-pure water system (Millipore Corporation, United States). Dry *S. baicalensis* and *B. chinense* were purchased from Inner Mongolia and An’hui was identified as *S. baicalensis* Georgi and *B. chinense* DC by Dr. Lihong Wu of Shanghai University of Traditional Chinese Medicine. Baicalin and Saikosaponin were purchased from Ciyuan Biotechnology (Shanxi, China).

### Animal Experiments and Samples Collection

All protocols for animal experimentation and maintenance were approved by the Animal Ethics Committee of Shanghai University of Traditional Chinese Medicine. Male Wistar rats (weighing 200 ± 10g) were provided with unrestricted amounts of food and water, housed in temperature- and humidity-controlled rooms, and kept on a 12-h light/dark cycle. One week later, all rats were divided into 8 groups of 8 rats each, randomized as follows: normal group, ALI modeling group and other six treatment groups. The ALI modeling groups were fed a high-glucose-fat diet (Hapon et al., 2005) (prescription: 10% cane sugar, 2% cholesterol, 10 % lard, 5 % yolk powder, 0.2% propylthiouracil, and 72.8% basal diet, purchased by Suzhou Shuangshi Laboratory Animal Feed Science, Suzhou, China), combined with 10 ml/kg liquor (Niemela et al., 1995) every 2 days for 30 days. The biochemistry indexes and histological results were employed to illustrate the successful modeling of ALI. All treatment groups were performed by intragastric administration, respectively, with decoctions of *B. chinense* (BU, amounting to 4 g/kg dried medicinal herbs), decoctions of *S. baicalensis* (SC, amount to 4 g/kg raw dried medicinal herbs), decoctions of *B. chinense-S. baicalensis* (BS, 1:1, amounting to 4 g/kg dried medicinal herbs), saikosaponin solution in water (the major active component of *B. chinense* referred to in Chinese Pharmacopeia (Lu et al., 2012) as SA, amounting to 4 g/kg dried medicinal herbs), baicalin solution in water [the major active component of *S. baicalensis* referred to in Chinese Pharmacopeia (Xi et al., 2015) as BA, amounting to 4 g/kg dried medicinal herbs], as well as the mixture of BA and SA (1:1, MI, amounting to 4 g/kg dried medicinal herbs) for 20 days. Additionally, normal and modeling rats were given equivalent double-distilled water.

After the modeling and treatment, the rats were placed in individual metabolic cages with fasting for 24 h to collect the urine and feces samples, which were kept at -80°C until analysis. Then, all the animals were anesthetized, and the serum samples were collected via abdominal aorta. All serum samples were centrifuged at 3,000 rpm at 4°C for 10 min. The supernatants were stored at -80°C for metabolomics and biochemical analysis. Liver tissues, dissected from the same lobe of each animal, were fixed in 4% paraformaldehyde overnight at room temperature, dehydrated through ethanol gradients, infiltrated in xylene, and embedded in paraffin. Serial sections of 5 μm thickness were made from paraffin-embedded tissue for hematoxylin and eosin (H&E) staining. Another set of liver samples, taken from the same lobe of each animal, were snap-frozen in liquid N_2_, and stored at -80°C.

### Biochemistry Indexes Analysis

Alanine aminotransferase, AST, GLU, CHOL, and TG were assayed by a Hitachi 7080 automatic biochemistry analyzer. All results were presented as the mean ± SD and analyzed using SPSS 17.0 (SPSS, Chicago, IL, United States). Multiple comparisons for two groups were performed by independent sample *T*-test. *P*-values below 0.05 were considered as statistically significant.

### Metabolomic Analysis

All serum, feces, and tissues were prepared for metabolomics analysis. For serum samples, the preparation was the same as in our previous reports (Sun et al., 2012; Dai et al., 2013b,c). For liver tissue, 50 mg of tissue was homogenized by 150 μL of normal saline. And for feces, 80 mg of fecal sample was transferred into a screw tube with 320 μL of normal saline and extracted with ultrasonic treatment for 20 min. Then, the derivatization process was performed as in our previous publications (Sun et al., 2012; Dai et al., 2013b,c).

All GC-MS analyses were performed by a mass spectrometer (5975B, Agilent, United States) coupled with a gas chromatography instrument (6890, Agilent, United States). The GC-MS operating condition was adapted from our previous reports (Sun et al., 2012; Dai et al., 2013b,c). The programmed column temperature was optimized to acquire a well separation; the column temperature was initially maintained at 70°Ñ for 2 min and then increased from 70 to 120°Ñ at a rate of 5°Ñ/min for 2 min. Then, the column temperature was increased to 190°Ñ at a rate of 3°Ñ/min, to 210°Ñ at a rate of 5°Ñ/min, to 260°Ñ at a rate of 10°Ñ/min. After that, the column temperature was increased to 290°Ñ at a rate of 5°Ñ/min and held for 3 min. The method of data analysis was also adapted from our previous reports (Sun et al., 2012; Dai et al., 2013b,c), optimizing the VIP values to 1.0 and the threshold of *P* value to 0.1. The variables that were discovered were identified by searching the NIST 2011 database and verified by standards. The Kyoto Encyclopedia of Genes and Genomes (KEGG) ^1^ and the Metabolites Biological Role (MBRole)^2^ were employed to highlight the most related pathway.

### Metallomic Analysis

Five hundred (500) μL of herbal extracts, 150 μL of serum or 100 mg of liver sample were removed into digestion tubes which were soaked with in 30% HNO_3_ at least 24 h followed by another 24 h in high purity water, and then rinsed again with high purity water. Three mL of 65% HNO_3_ was added into the tubes and these tubes were then put into an airing chamber for 12 h. The samples carried out microwave digestion with the different temperature program (Supplementary Data Sheet 1 for details). Subsequently, these solutions were transferred into 50 mL centrifuge tubes and the tubes were washed with high ultrapure water three times to ensure that the concentration of HNO_3_ was less than 5%. All these solutions were stored at 4°C (Mesko et al., 2011), and awaited analysis by ICP-MS.

Environmental calibration standard solutions—10 mg/L^-1^ of Al, As, Ba, Be, Co, Cr, Cu, Mn, Ni, Pb, Se, V, and Zn, in addition to 1000 mg/L^-1^ of Ca, Fe, K, Mg, and Na—were diluted into a series of concentrations with 5% HNO_3_.

Before ICP-MS analysis, it was necessary to be purged by helium (99.99%) at 2 L/min. When the forward power reached 1500 W and the reflected power reached 1 W, it indicated that the system could ignite to start up. The experimental parameters used for ICP-MS analysis are summarized in Supplementary Data Sheet 1. Furthermore, calibration curve, LLOQ (the lower limit of quantification), precision test and repeated experiment were carried out to validate the method reliability.

All results were presented as the mean ± SD and analyzed using SPSS 17.0 (SPSS, IBM, United States). Multiple comparisons for two groups were performed by independent sample *T*-test. A *P* value less than 0.05 was considered statistically significant.

## Results

### Pharmacodynamic Evaluation of *Bupleurum*-*Scutellaria* and Its Compositions

**FIGURE 1:**
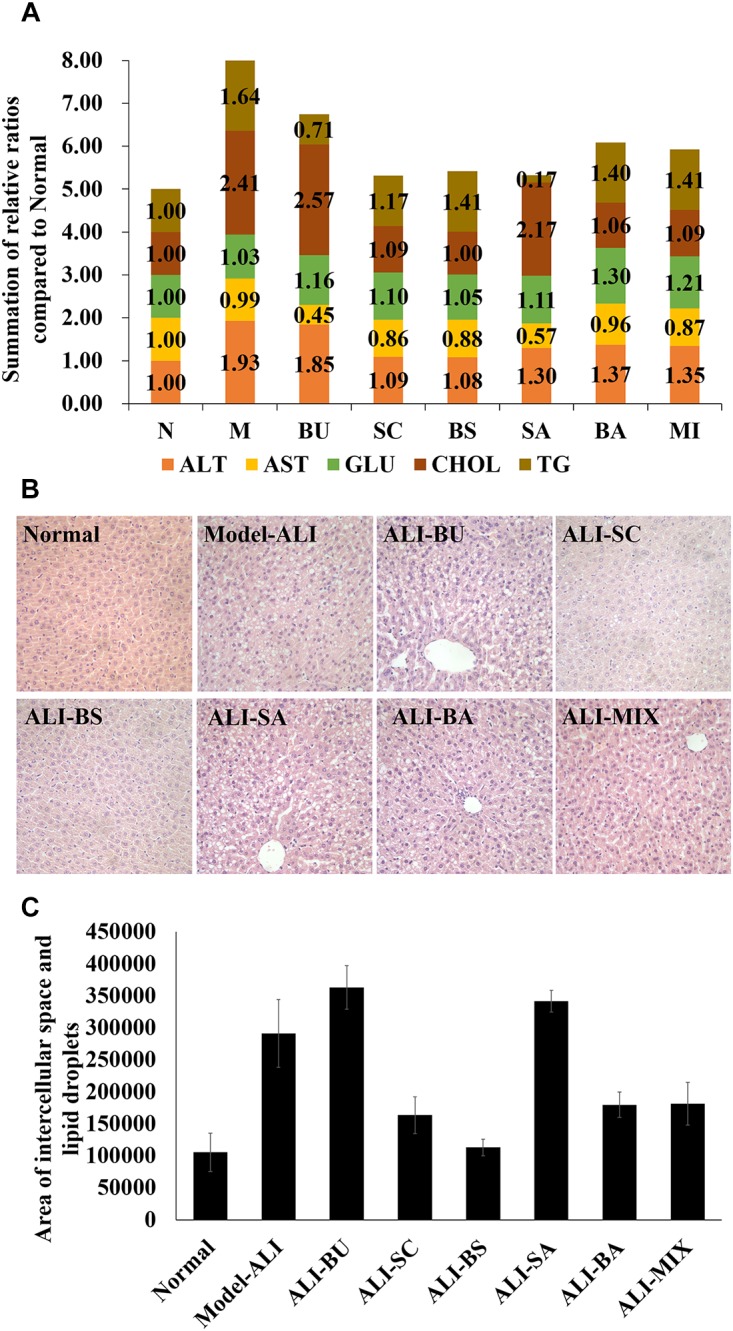
Biochemical and histological analysis. **(A)** Biochemical analysis of major biomarkers for liver functions in blood of rates. The biochemical indicators of *Bupleurum chinense-Scutellaria baicalensis* and their composition were compared to the normal control group; **(B)** liver tissue histopathology (H&E staining) of every group; **(C)** the quantized results of liver tissue histopathology. The results were based on the area of intercellular space and lipid droplets to present liver cell swelling and lipid droplets accumulation. N: Normal control; M: Alcoholic Liver Injury Modeling; ALI: Alcoholic Liver Injury; BS: decoctions of *B. chinense-S. baicalensis*; BU: decoctions of *B. chinense*; SC: decoctions of *S. baicalensis*; SA: water solution of Saikosaponin; BA: water solution of Baicalin; MI: the mixture water solution of Baicalin and Saikosaponin; Alanine aminotransferase (ALT), Aspartate aminotransferase (AST), Glucose (GLU), Cholesterol (CHOL), Triglyceride (TG).

First, biochemistry indexes and histological results were employed to illustrate pharmacodynamic effects of *B. chinense-S. baicalensis* and its compositions. The biomarkers, including ALT, AST, GLU, CHOL, and TG, which reflect the liver-associated disorder, were used to evaluate the physiological changes with treatment of BS and its compositions in ALI (detail in Supplementary Table S1). We found that decoctions of *B. chinense-S. baicalensis* and *S. baicalensis*, alongside a water solution of Baicalin and in addition to the mixture of Baicalin and Saikosaponin, could reverse the biochemical disturbances in ALI (Figure 1A,B). However, *B. chinense* and its major active component, Saikosaponin, present little pharmacodynamic effects (Figure 1A,B). Then, the histological results evaluated the improvement of pathological conditions. Some liver injury characteristics, such as liver cell swelling, lipid droplets accumulation, and inflammatory infiltration occurred in the ALI model group (Figure 1C). It is worth pointing out that *B. chinense-S. baicalensis* nearly reversed all ALI-induced pathological conditions to normal (Figure 1C). The decoctions of *S. baicalensis* showed slightly worse reversion than *B. chinense-S. baicalensis* in all ALI-induced pathological changes. Treatment of Baicalin and the mixture of Baicalin and Saikosaponin could significantly reduce lipid droplets accumulation (Figure 1C). Furthermore, we found that *B. chinense* and Saikosaponin did not present improvement of liver injury characteristics (Figure 1C), which was consistent with the results of biochemistry indexes. So, it seems that SC and BA may, respectively, play an important role in BS and MI’s treatment efficacy for ALI. More importantly, these results provided evidence of the synergy, more exact curative effect, in BS treatment in the ALI group at both the biochemical and histopathological levels. Why it happened? How were these emergent characteristics brought out? The above questions prompted us to investigate the potential mechanism by which the efficacy of *B. chinense-S. baicalensis*, rather than the single herbs and their active ingredients, was enhanced.

### Systematic Analysis of Drug-Induced Metabolic Perturbations

**FIGURE 2:**
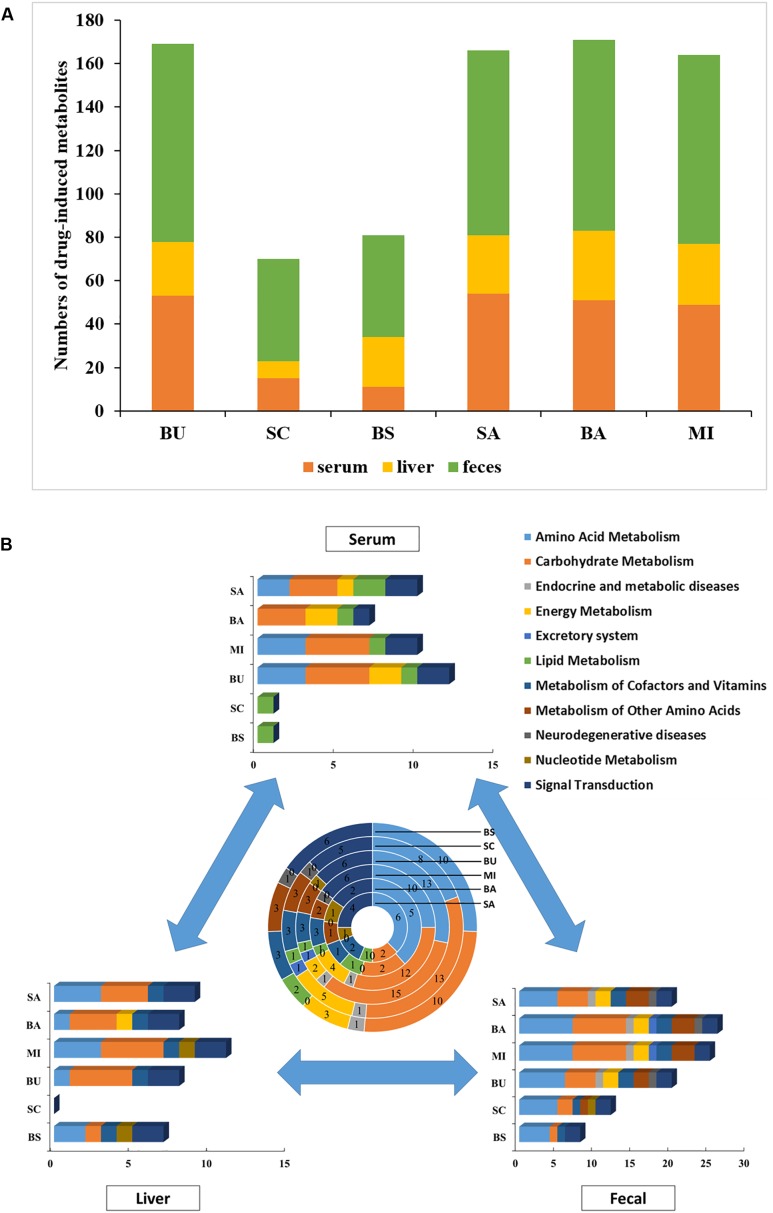
Metabolite and pathway analysis of drug-induced perturbations. **(A)** Numbers of drug-intervened metabolites in ALI; **(B)** numbers of drug-intervened pathways in ALI. The summarization of drug-intervened global, local and symbiotic pathways is presented in the center. BS: decoctions of *B. chinense-S. baicalensis*; SC: decoctions of *S. baicalensis*; BU: decoctions of *B. chinense*; BA: water solution of baicalin; SA: water solution of saikosaponin; MI: the mixture of baicalin and saikosaponin.

Compared with the ALI group, the metabolites of serum, liver tissue, and fecal extracts, disturbed by BS or its components, were employed to construct global, local, and symbiotic metabolic profiling (Figure 2A), respectively. First, we analyzed the metabolites that only changed in BS treatment (Table 1 and Supplementary Table S2). After preliminary structural and functional analysis, we found that unique BS-intervened metabolites were enriched in alpha amino acids and derivatives (serine, phenylalanine and ornithine) and aliphatic acyclic compounds (urea and methanamine), which are involved in ATP-binding cassette transporter (urea, phenylalanine, ornithine and serine), Microbial metabolism in diverse environments (methanamine, urea and serine) and biosynthesis of amino acids (phenylalanine, ornithine and serine).

**Table 1:** The special metabolites detected in BS group of ALI compared with control group.

Compound	Location	VIP^a^	FN^b^	*p*-value^c^
1,3-Butadiene	Serum	1.53	1.67	0.07
Pregnenolone	Serum	1.50	1.67	0.07
Serine	Serum	1.51	0.49	0.02
Pyrazofurin	Serum	1.48	0.39	0.01
Urea	Serum	2.09	2.34	0.01
beta.-D-Galactofuranoside	Feces	1.21	0.49	0.02
L-Norleucine	Feces	1.56	2.02	0.02
Ornithine	Feces	1.66	2.24	0.01
Pteridine	Feces	1.32	1.96	0.02
Thiadiazolidinone	Feces	1.06	1.89	0.03
D-Glucuronic acid	Feces	1.08	1.87	0.04
Methanamine	Feces	1.49	1.87	0.04
Phenylalanine	Feces	1.20	0.48	0.04
Pyrimidine	Liver	1.55	0.43	0.01
Uridine	Liver	1.32	1.96	0.02


*^a^VIP, variable importance in the project; ^*b*^FN is fold change of mean ranks calculated by the Mann-Whitney test (BS compared with control group). *p*-Value was obtained from Student’s *t*-test (BS compared with control group).*

However, these metabolites could not provide the mechanisms needed to unveil BS’s unique efficacy. All drug-disturbed metabolites were, respectively, enriched into pathways by MBRole, which were further categorized into functional modules by KEGG (Figure 2B). Surprisingly, we found that the numbers of changed serum metabolites in the BS group were the least among all groups (Figure 2A). According to the results of biochemical indicators and pathology, we presumed that BS’s unique efficiency may be derived from the precise regulation of disease-intervened metabolites with less bypass perturbations. Further metabolic analysis could provide the evidence for this hypothesis. The symbiotic bacteria metabolism was disturbed by BS group most slightly but amino acid metabolism, carbohydrate metabolism and signal transduction. Furthermore, it was observed BS in serum-metabolite profiling only intervened in lipid metabolism, and SC may play a major role in this pathway. However, SC could not intervene in liver metabolism but BU could. We hypothesized that BS largely exerted its prefect effects through tissue penetration with the effect of BU. Integrating the global, local and symbiotic perspective, striking differences were observed in the BS group compared with other groups, such as more effects in amino acid metabolism, nucleotide metabolism and signal transduction but less disturbances in other disease-related metabolism (such as endocrine and metabolic diseases as well as neurodegenerative diseases). Finally, we were eager to find out the reasons for which usage of the herbal pair could provide beneficial pharmacodynamic effects.

### Diversity of Metal Elements in Serum and Liver Tissue May Derive From Better Pharmacodynamic Effects of Herbal Pair

Preliminarily, we wanted to explain the mechanism of the formation of new compounds, but previous research proved that there were not more constituents from chemical transformation (Dai Peihui et al., 2017). As even the major active components (Baicalin and Saikosaponin) could not bring better efficacy (Figure 1), we changed our focus to the metal elements with many physiological and biochemical effects related to the pharmacodynamic mechanism (Dong et al., 2006; Yan et al., 2015; Deng et al., 2018) of TCM. Metallomics was employed to explore diverse metal elements in the herbal extraction, serum and liver tissue. This approach could help us to illustrate the combined effects of the *B. chinense-S. baicalensis* herbal pair.

**FIGURE 3:**
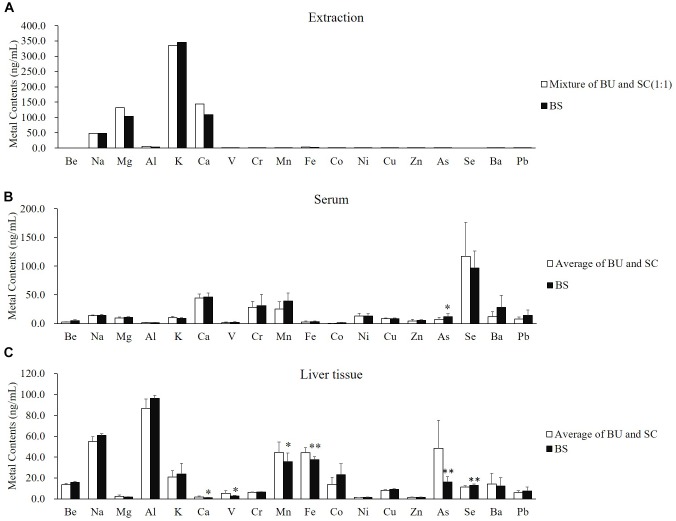
*B. chinense*-*S. baicalensis* herbal pair altered the distribution of metal contents in the serum and liver tissue. **(A)** Comparison of metal contents between average of BU and SC (white column) and BS (black column). **(B)** Comparison of serum metal contents between average of BU-treated and SC-treated rats (white column) and BS-treated rats (black column). **(C)** Comparison of hepatic metal contents between average of BU-treated and SC-treated rats (white column) and BS-treated rats (black column). BU: decoctions of *B. chinense*; SC: decoctions of *S. baicalensis*; BS: decoctions of *Bupleurum chinense*-*S. baicalensis*.

We found that there was no significant difference at the comparation of metal contents between the average of BU and SC (white column) and BS (black column) (Figure 3A). The different distributions of metal elements in the serum and liver tissue were not because of the distinguished input of mental elements. Interestingly, the distributions of elements were obviously different between the serum and liver tissue. In the serum, most elements presented no differences between the average of BU-treated and SC-treated rats (white column) and BS-treated rats (black column), except arsenic (Figure 3B). However, in liver tissue, calcium, vanadium, manganese, iron, and arsenic were significantly reduced by treatment of BS, while the content of selenium was clearly increased (Figure 3C). Selenium particularly caught our attention because it was not detected in the extraction of BU, SC, and BS (Figure 3A). These results suggested that BS significantly regulated the distribution of endogenous selenium in liver tissue. As selenium plays an important role in the anti-oxidative and hepatoprotective effects, it may be the reason behind the combined effects of the BS formula.

## Discussion

The TCM formula, which is composed of related herbs under the theories of TCM, has been applied in clinical treatment to provide more efficiency and safety. There is a trend of combination therapy to achieve combined effects in complex diseases, such as breast cancers (Hyung et al., 2011), liver fibrosis (Zhang H. et al., 2016). However, it is challenging to explore the combined effect of more complex TCM with deeper insight. To elucidate the combined mechanisms of the TCM formula, researchers have undertaken much research. The “Realgar-Indigo naturalis formula,” which was composed of Realgar, Indigo naturalis and *Salvia miltiorrhiza*, has been proven to be very effective in treating human acute promyelocytic leukemia promyelocytic leukemia. Three major active ingredients (tetraarsenic tetrasulfide, indirubin, and tanshinone IIA are the major active ingredients in Realgar, Indigo naturalis and *Salvia miltiorrhiza*, respectively) yielded synergy in the treatment of acute promyelocytic leukemia *in vivo* and *in vitro*. They dissected the mechanisms of TCM formulae at the molecular, cellular and organismal levels (Wang et al., 2008). There have also been other examples, such as Shuang-Huang-Lian (Feng et al., 2005; Di et al., 2006), Yin-Chen-Hao-Tang (Hyung et al., 2011; Wang et al., 2011) and Wen-Xin-Formula (Dai et al., 2013b), yet systematic evaluation for the pharmacodynamic effects and the material basis of the combined effect have not been well illustrated.

In this study, we employed biochemical and pathological analyses combined with metabolomics to provide the system evaluation for the pharmacodynamic effects of the *B. chinense-S. baicalensis* herbal pair, the single herbs and their active ingredients. In our study, we found that BS clearly intensified the therapeutic efficacy on both biochemical and histological levels. These observations provided insight into the combined effect. As analysis of biochemical and pathological results, we could claim that *S. baicalensis* and its major active compound, baicalin, may play important roles to ameliorate lipid droplet accumulation, as observed in the *B. chinense-S. baicalensis* herbal pair group. Some research that showed that baicalin presented antisteatotic effects also support our results (Guo et al., 2009; Kim and Lee, 2012; Xi et al., 2015). Though lipid metabolism disorder is one of the most notable characteristics in ALI, improvement of cell swelling and inflammatory infiltration gradually diminished form the herbal pair to single herb and baicalin (Figure 1). This may suggest that other components in *S. baicalensis* such as baicalein (Fan et al., 2013; Patwardhan et al., 2016), wogonin (Lin and Shieh, 1996; Lee et al., 2015) and oroxylin A (Shimizu et al., 2018), showed the main anti-inflammatory and hepatoprotective activity. In contrast to our expectation, *B. chinense* and saikosaponin may not be able to cure ALI (Figure 1). However, the metabolomic analysis of liver indicated that *S. baicalensis* hardly intervened in the metabolomic pathways in liver tissue (Figure 2B). Yet, *B. chinense-S. baicalensis* could significantly enhance tissue penetration and influenced the pathways of intestinal flora. This may address one of the common problems in combination therapy, diarrhea. All these results suggested that the *B. chinense-S. baicalensis* herbal pair presented better effects in the treatment of ALI than single herbs and their active compounds.

To reveal the connotation of the formula, it is necessary to understand not only the main ingredients but also the combined application of multilevel analytical and synthetic research approaches at the molecular, cellular, and organismal levels. In our case, we paid attention to metal elements, as previous research had proved that only the changes of baicalin and saikosaponin (Dai Peihui et al., 2017) could not contribute to the better efficacy in our results (Figure 1). After metallomic analysis of BS herbal pair treatment, we found that arsenic was increased in the serum, but calcium, vanadium, manganese, iron and arsenic were significantly decreased in liver tissue, while contents of selenium were increased in liver tissue (Figure 3B,C). The results revealed that BS herbal pair treatment could reduce toxic metal accumulation in the liver, such as vanadium (Hosseini et al., 2013), manganese (Fujimura et al., 2012), iron (Zhang et al., 2014) and arsenic (Tan et al., 2011; Massey et al., 2015). The arsenic in the serum could intensify the expression of aquaglyceroporin and enhance PML-RAR degradation and anti-inflammatory efficacy (Wang et al., 2008). At the same time, selenium in the liver could significantly inhibit adipocyte hypertrophy and abdominal fat accumulation via induction of fatty acid beta-oxidation (de Castro Barra et al., 2015) and alter lipid metabolism and protein synthesis in the liver (Zhao et al., 2016). Furthermore, selenium could be incorporated into selenoproteins which play important roles in the anti-oxidative stress and hepatoprotective effect via apoptosis inhibition (Tang et al., 2018). The selenoproteins could participate in pyrimidine metabolism (Carlson et al., 2007), which is the unique pathway of intervention by the BS herbal pair in liver tissue (as shown in Table 2). Thus, the transition from pyrimidine to uridine was increased (as shown in Table 2). The enhanced uridine homeostasis-linked liver pyrimidine metabolism could ameliorate lipid accumulation (Le et al., 2013). Furthermore, simultaneously increased contents of selenium and uridine could show a cytoprotection effect through enhancing the availability of precursors and cofactors in the circulation (Rijpma et al., 2015). Finally, selenium could reverse drug-induced immunotoxic effects (Latorre et al., 2011) to relieve drug pressure. In summary, the elevated selenium may be one of the potential causes that lead to enhancing efficacy and reducing toxicity in the *B. chinense-S. baicalensis* herbal pair.

**Table 2:** Contributions of the single herbs and their active ingredients to the disturbed pathways of *B. chinense-S. baicalensis* herbal pair.

Source	Pathway	Constituents				
		*Scutellaria baicalensis*	*Bupleurum chinense*	Baicalin	Saikosaponin	Mixture
Serum	Biosynthesis of unsaturated fatty acids	✓	✓	✓	✓	✓
Liver	ABC transporters		✓	✓	✓	✓
	Aminoacyl-tRNA biosynthesis		✓	✓	✓	✓
	Pantothenate and CoA biosynthesis		✓	✓	✓	✓
	Propanoate metabolism		✓	✓	✓	✓
	Pyrimidine metabolism					✓
	Valine, leucine and isoleucine biosynthesis		✓	✓	✓	✓
	Valine, leucine and isoleucine degradation				✓	✓
Feces	ABC transporters	✓	✓	✓	✓	✓
	Aminoacyl-tRNA biosynthesis	✓	✓	✓	✓	✓
	Cysteine and methionine metabolism	✓	✓	✓	✓	✓
	Pantothenate and CoA biosynthesis	✓	✓	✓	✓	✓
	Phenylalanine metabolism	✓	✓	✓	✓	✓
	Phenylalanine, tyrosine and tryptophan biosynthesis	✓	✓	✓	✓	✓
	Propanoate metabolism	✓	✓	✓		✓
	Valine, leucine and isoleucine biosynthesis	✓	✓	✓	✓	✓


## Conclusion

**FIGURE 4:**
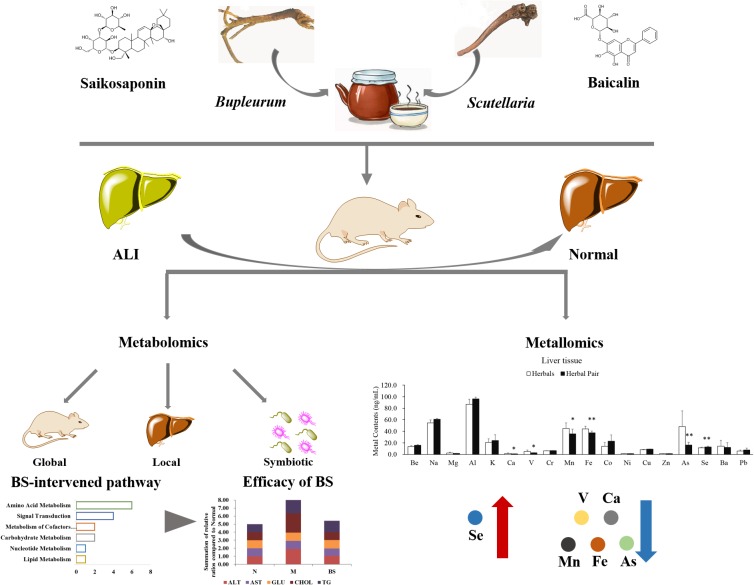
Schematic diagram of the combined effects of the *B. chinense-S. baicalensis* herbal pair in Alcoholic Liver Injury. Alcoholic Liver Injury was used to research the characteristics of *B. chinense- S. baicalensis*, which is controlled by *B. chinense* and *S. baicalensis*, and their main active ingredients baicalin and saikosaponin. The biochemical indicators and histology indicated that *B. chinense-S. baicalensis* presented the best efficacy, which could be further illustrated by global, local, and symbiotic metabolic analysis. Finally, metallomic analysis was employed to uncover the distribution changes of selenium which, unlike other metal elements, was significantly increased by *B. chinense-S. baicalensis* in liver tissue to demonstrate multiple benefits such as anti-oxidative stress and hepatoprotective effect, to relieve drug pressure.

In this study, we conducted an animal model of ALI to study the characteristics of the *B. chinense-S. baicalensis* herbal pair, which was controlled by *B. chinense*, *S. baicalensis*, and their main active ingredients baicalin and saikosaponin. Biochemical and histological indicators revealed that the effect of *B. chinense-S. baicalensis* is the best in all treatment groups. Furthermore, metabolite profiling of the serum, liver tissue and feces was conducting with GC-MS to comprehensively reveal the global, local and symbiotic metabolic modulation. Our results indicated that BS largely developed its effects through enhanced tissue penetration to maintain liver-located intervention with less global and symbiotic disturbance. Furthermore, we analyzed the distribution of the metal elements in the serum and liver tissue in *B. chinense-S. baicalensis*, and found that selenium was significantly increased in liver tissue. The increased selenium may show multi-beneficial effects for the treatment efficacy in *B. chinense-S. baicalensis*. The schematic diagram of the combined effects of the *B. chinense-S. baicalensis* herbal pair in ALI is shown in Figure 4. This observation preliminarily showed the combined effects in the *B. chinense-S. baicalensis* herbal pair, which may provide a new perspective for exploring the material basis of combined effects in natural herbal medicine, especially TCM.

## Author Contributions

YZ and JD contributed to conception and design of the study. NZ, QL, SS, YW, ZD, and JD organized the database. NZ, QL, SS, YW, ZD, and JD performed the statistical analysis. JD wrote the first draft of the manuscript. NZ, QL, SS, YW, ZD, and YZ wrote sections of the manuscript. All authors contributed to manuscript revision, read and approved the submitted version. All authors have given approval to the final version of the manuscript.

## Conflict of Interest Statement

The authors declare that the research was conducted in the absence of any commercial or financial relationships that could be construed as a potential conflict of interest.

## Footnotes

1 www.genome.ad.jp/kegg/

2 http://csbg.cnb.csic.es/mbrole2/

## Abbreviations

ALI: alcoholic liver injury
ALT: alanine aminotransferase
AST: aspartate aminotransferase
BA: baicalin
BS: *Bupleurum chinense-Scutellaria baicalensis*
BU: *Bupleurum chinense*
CHOL: cholesterol
GLU: glucose
OPLS: orthogonal partial least squares
PCA: principle component analysis
PLS-DA: partial least squares-discriminant analysis
SA: saikosaponin
SC: *Scutellaria baicalensis*
TCM: traditional chinese medicine
TG: triglyceride
