# Supplementary material for: The Medicinal Plant Pair Bupleurum chinense-Scutellaria baicalensis – Metabolomics and Metallomics Analysis in a Model for Alcoholic Liver Injury
Source: Front Pharmacol. 2019 Mar 20;10:254. doi: 10.3389/fphar.2019.00254 (PMC6445227; doi:10.3389/fphar.2019.00254)
Supplement: Supplementary file 3 [file Data_Sheet_1.pdf]

Sheet 1 Experimental parameters for ICP-MS and GC-MS

a The experimental parameters used for ICP-MS

Parameters	
Forward Power	1500 W
Reflected Power	1 W
Carrier Gas	1.0 L/min
S/C Temperature	2 °C
Nebulizer Pump	0.1 rps
Repetition	3
Uptake Speed	0.30 rps
Uptake Time	30 sec
Stabilization Time	45 sec

b The temperature program of GC-MS

Rate(°C/min)	Temperature(°C)	Hold time(min)
	70	2
5	120	2
3	190	0
5	210	0
10	260	0
5	290	0
